# Leveraging large language models for automated depression screening

**DOI:** 10.1371/journal.pdig.0000943

**Published:** 2025-07-28

**Authors:** Bazen Gashaw Teferra, Argyrios Perivolaris, Wei-Ni Hsiang, Christian Kevin Sidharta, Alice Rueda, Karisa Parkington, Yuqi Wu, Achint Soni, Reza Samavi, Rakesh Jetly, Yanbo Zhang, Bo Cao, Sirisha Rambhatla, Sri Krishnan, Venkat Bhat

**Affiliations:** 1 Interventional Psychiatry Program, St. Michael’s Hospital, Unity Health Toronto, Toronto, Ontario, Canada; 2 Electrical and Computer Engineering, University of Toronto, Toronto, Ontario, Canada; 3 Department of Mathematical and Computational Sciences, University of Toronto, Toronto, Ontario, Canada; 4 Electrical and Computer Engineering, University of Alberta, Edmonton, Alberta, Canada; 5 Department of Computer Science, University of Waterloo, Waterloo, Ontario, Canada; 6 Electrical, Computer, and Biomedical Engineering, Toronto Metropolitan University, Toronto, Ontario, Canada; 7 Institute of Mental Health Research, University of Ottawa, Ottawa, Ontario, Canada; 8 Department of Psychiatry, Faculty of Medicine and Dentistry, University of Alberta, Edmonton, Alberta, Canada; 9 Ministry of Health, Government of Alberta, Edmonton, Alberta, Canada; 10 Department of Computing Science, University of Alberta, Edmonton, Alberta, Canada; 11 Department of Psychiatry, University of Toronto, Toronto, Ontario, Canada; Massachusetts Institute of Technology, UNITED STATES OF AMERICA

## Abstract

Mental health diagnoses possess unique challenges that often lead to nuanced difficulties in managing an individual’s well-being and daily functioning. Self-report questionnaires are a common practice in clinical settings to help mitigate the challenges involved in mental health disorder screening. However, these questionnaires rely on an individual’s subjective response which can be influenced by various factors. Despite the advancements of Large Language Models (LLMs), quantifying self-reported experiences with natural language processing has resulted in imperfect accuracy. This project aims to demonstrate the effectiveness of zero-shot learning LLMs for screening and assessing item scales for depression using LLMs. The DAIC-WOZ is a publicly available mental health dataset that contains textual data from clinical interviews and self-report questionnaires with relevant mental health disorder labels. The RISEN prompt engineering framework was utilized to evaluate LLMs’ effectiveness in predicting depression symptoms based on individual PHQ-8 items. Various LLMs, including GPT models, Llama3_8B, Cohere, and Gemini were assessed based on performance. The GPT models, especially GPT-4o, were consistently better than other LLMs (Llama3_8B, Cohere, Gemini) across all eight items of the PHQ-8 scale in accuracy (M = 75.9%), and F1 score (0.74). GPT models were able to predict PHQ-8 items related to emotional and cognitive states. Llama 3_8B demonstrated superior detection of anhedonia-related symptoms and the Cohere LLM’s strength was identifying and predicting psychomotor activity symptoms. This study provides a novel outlook on the potential of LLMs for predicting self-reported questionnaire scores from textual interview data. The promising preliminary performance of the various models indicates there is potential that these models could effectively assist in the screening of depression. Further research is needed to establish a framework for which LLM can be used for specific mental health symptoms and other disorders. As well, analysis of additional datasets while fine-tuning models should be explored.

## Author summary

Mental health diagnoses present unique challenges that may affect well-being and daily functioning. To aid in screening, self-report questionnaires, such as the PHQ-8, are widely used but rely heavily on subjective responses. Patient responses to these questionnaires are often influenced by external factors, personal beliefs, and response bias. Leveraging advancements in large language models (LLMs), we sought to demonstrate an automated predictive model for assessing depression item scales using textual interview data. Utilizing the DAIC-WOZ dataset, which includes clinical interview transcripts and self-report questionnaire responses, we employed the RISEN prompt engineering framework to evaluate LLMs’ ability to predict individual PHQ-8 items. Our analysis compared models, such as GPT-4o, Llama 3-8B, Cohere, and Gemini. GPT models excelled, achieving a higher average accuracy and F1 score, with promising performance in emotional and cognitive symptoms. Llama outperformed others in detecting anhedonia, while Cohere excelled at psychomotor activity prediction. These findings suggest that LLMs may have the potential for clinical integration to enhance mental health screening.

## Introduction

Mental health disorders are prevalent and often underdiagnosed conditions that can significantly impact an individual’s well-being and daily functioning [1]. Disorders including, but not limited to, depression, anxiety, insomnia, schizophrenia, and bipolar disorder pose substantial challenges for individuals and the healthcare system which can often lead to misdiagnoses [2,3]. Depression is one of the leading causes of disability worldwide [4]. The complex and multidimensional nature of public opinion and stigma towards mental health disorders presents barriers in an individual’s perception and open presentation of their experiences [5]. While each disorder presents nuanced challenges in its management and treatment, effective and timely diagnosis is critical for improving patient outcomes and encouraging an effective method of societal integration [6].

Early diagnosis and the creation of effective personalized treatment plans can often be challenging when considering mental health disorders due to the subjective nature and potential overlap of reported symptoms [7]. In clinical practice, self-report questionnaires are commonly distributed as a method to screen for mental health disorders and their severity. Clinical assessments that rely on respondents’ subjective reports (e.g., Patient Health Questionnaire (PHQ) for depression [8], Generalized Anxiety Disorder 7-item scale (GAD-7) for anxiety [9]) have become regular fixtures in assessing mental health disorders [10]. Despite being a valuable standard practice in healthcare, self-reported responses can be influenced by several factors [11]. For example, mental health self-awareness or societal stigma surrounding mental health has been shown to change the quality of self-reporting, including a reluctance to disclose personal information [11]. These influences can potentially lead to inaccuracies in the overall assessment of the disorder and its severity, which could negatively impact the effectiveness of treatment planning [12]. However, self-report measures allow patients to describe their own experiences which observational data may not capture. Therefore, it is important to explore additional tools that can supplement self-reported questionnaire data while mitigating potential response bias.

The arrival of, and increasing research on, large language models (LLMs) presents a promising opportunity to improve the screening of mental health disorders [13,14]. An LLM is an AI model capable of generating and processing natural human language patterns based on neural networks and extensive training on large textual datasets. Vast amounts of textual data (e.g., social media posts [15], electronic health records [16], interviews [17]) can be used to train LLMs which can enhance capabilities in understanding and producing human-like speech patterns (e.g., idea organization, tone, contextual information) [15–18]. Machine learning systems that use supervised learning strategies (training that involves labelled datasets) and feature extraction methods can use data paired with the correct “label” to subsequently improve their own performance [19]. However, deep-learning-based approaches, such as supervised learning, require a large number of training data to obtain a good diagnostic model. LLMs do not require large amounts of labelled data to make predictions due to their existing knowledge base before a prompt [20]. For example, many LLMs are trained on large, diverse sets of unlabeled text data. They can perform zero-shot learning, allowing them to generalize to new tasks or concepts without task-specific training on labelled examples.

LLMs also offer distinct advantages for predicting depression scale compared to traditional machine learning (ML) and Natural Language Processing (NLP) approaches. Unlike classical ML models that rely on handcrafted linguistic features or lexicon-based sentiment analysis, LLMs can capture subtle linguistic cues such as implicit distress, tone, and contextual meaning [21], which are critical for assessing depressive symptom severity. Additionally, LLMs demonstrate strong generalization capabilities, allowing them to adapt to diverse linguistic expressions of depression without the need for extensive domain-specific tuning [22]. LLM-based methods provide a promising alternative by enabling automated, scalable, and context-aware analysis of language, which may improve the accuracy and accessibility of depression screening.

While self-report instruments such as the PHQ-8 are valuable for assessing internal states, they require active engagement and may be underutilized in many populations. LLMs, trained on rich subjective language data, offer the potential to passively infer similar mental health constructs without the need for structured assessment. However, we do not suggest that LLMs overcome the subjectivity inherent in self-report data. Rather, our aim is to evaluate whether models trained on verbal expressions of internal experience can reasonably approximate structured survey scores, which are themselves subjective but standardized. Previous research has demonstrated that there is a correlation between the linguistic features of speech and the presence of anxiety allowing for transformer-based neural networks to predict the presence of anxiety from speech transcripts alone [21,23].

Incorporating LLMs into healthcare research can also offer a scalable solution in various digital health platforms. By streamlining tasks like symptom detection and mental health screening, LLMs have the potential to bridge gaps in healthcare accessibility and improve outcomes, particularly in underserved or resource-limited settings. Furthermore, their ability to integrate seamlessly with existing digital tools makes them good candidates for enhancing telemedicine platforms, virtual therapy solutions, and remote monitoring systems.

There has been a notable increase in LLM research in recent years, leading to a variety of LLMs being developed and validated. Each LLM was designed to process and generate human speech patterns but with unique differences in architecture, training data, and intended uses. One example is the GPT models which were developed by OpenAI in 2022, with ongoing upgrades and advancements. These GPT models are designed to generate human-like text and perform tasks using nuanced language techniques. While Llama, an open-source LLM created by Meta AI, was recently developed to be highly efficient in resource management while upholding comparable efficiency to GPT models. As well, Cohere is another significant LLM that emphasizes customization for specific applications to suit specific needs. Different LLM models, despite similar goals, must be robustly compared to uncover aspects of system performance metrics and feasibility/accessibility within healthcare environments.

This study details the methodology used to develop and evaluate LLM-based prediction models of individual depression symptoms using a publicly available dataset titled ‘Distress Analysis Interview Corpus/Wizard-of-Oz’ (DAIC-WOZ). Focusing on textual interview transcripts relevant to mental health disorder outcomes, we aim to evaluate the accuracy and performance of out-of-box LLMs using zero-shot learning in predicting individual symptom scores to enhance the understanding and potential of incorporating LLMs in mental health assessments and predictions. To our knowledge, this is the first study of its kind to evaluate and compare a variety of different LLMs in screening for depression by individually considering the different depressive symptoms.

The objective of the study is to determine the suitability of using LLM to provide a standardized scoring framework for self-reported mental health questionnaires. This will be achieved by a methodology consisting of the following steps: (1) identify and acquire publicly available mental health datasets that contain textual data and corresponding mental health disorder labels, (2) pre-process and clean the selected dataset to make it suitable for input into LLMs, (3) employ prompt engineering techniques to adapt different LLMs for the task of predicting mental health disorder sub-item labels from textual data, and (4) evaluate the performance of out-of-box LLMs in predicting depression symptoms as marked by individual questionnaire items.

## Results

We evaluated the performance of seven models from four out-of-the-box LLM platforms (GPT, Cohere, Llama, and Gemini) on 100 randomly selected interview sessions from the DAIC-WOZ dataset, considering both the original Likert rating scale and the binarized form of the items. The results for each individual questionnaire item, and the average model performance across all eight questionnaire items, are presented for the evaluated LLM models. The performance metrics used to assess the models were Accuracy rate, F1-score, and Matthews Correlation Coefficient (MCC).

### Average model performance

As shown in Table 1, GPT-4o performed with the highest mean accuracy from all eight PHQ8 items, for both Likert rating scale scoring (*M* = 48.9%, *SD* = 11.0) and binarized scoring (*M* = 75.9, *SD* = 6.8). The GPT-4o model’s high performance on F1 score (Likert scale scoring: *M* = 0.43, *SD* = 0.12; binary scoring: *M* = 0.74, *SD* = 0.1) and MCC metrics (Likert scale scoring: *M = *0.23, *SD* = 0.10; binary scoring: *M* = 0.25, *SD* = 0.13) underscores its balanced and reliable predictions. GPT 4 model variants consistently outperformed the GPT-3.5 model. In the Likert scoring system, the GTP-4o Mini had the best F1-score out of all the GPT models (M = 0.44, SD = 0.10) however, GPT-4o had the higher accuracy, precision, recall, and MCC (Table 1).

**Table 1:** Descriptive statistics (mean and standard deviation) for the 7 LLMs of interest across Likert and binary scoring systems.

Likert Scoring System (0 – 3)					
Model	Precision *M ± SD*	Recall *M* ± SD	Accuracy *M ± SD*	F1-score *M* ± SD	MCC *M* ± SD
**Cohere**	0.49 **± **0.08	0.47 **± **0.11	46.5 **± **10.6	0.41** ± **0.11	0.20 **± **0.08
**Gemini**	0.47 **± **0.08	0.47 **± **0.07	46.5 **± **7.8	0.44** ± **0.09	0.17 **± **0.12
**Llama 3_8B**	0.40 **± **0.08	0.46 **± **0.10	46.1** ± **10.3	0.40 **± **0.10	0.14 **± **0.09
**GPT-3.5**	0.42 **± **0.09	0.44** ± **0.11	44.4 **± **10.9	0.39 **± **0.11	0.16 **± **0.08
**GPT-4o Mini**	0.46 **± **0.11	0.48** ± **0.09	47.5 **± **9.3	**0.44 ± 0.10**	0.18** ± **0.12
**GPT-4o**	**0.49 ± 0.09**	**0.49 ± 0.11**	**48.9 ± 11.0**	0.43 **± **0.12	**0.23 ± 0.10**
**GPT-4 Turbo**	0.43 **± **0.13	0.46 **± **0.12	45.8 **± **12.0	0.40 **± **0.13	0.14** ± **0.13
**Binary Scoring (0 or 1)**					
**Model**	**Precision** ***M ± SD***	**Recall** ***M* ± SD**	**Accuracy** ***M ± SD***	**F1-score** ***M *± SD**	**MCC** ***M* ± SD**
**Cohere**	0.76** ± **0.07	0.75** ± **0.08	75.0 **± **7.9	0.73 **± **0.1	**0.35 ± 0.09**
**Gemini**	0.74** ± **0.08	0.72 **± **0.09	72.3 **± **8.7	0.7 **± **0.1	0.28** ± **0.16
**Llama 3_8B**	0.76** ± **0.05	0.76** ± **0.08	75.5 **± **7.8	0.73 **± **0.1	0.31** ± **0.15
**GPT-3.5**	0.76** ± **0.06	0.73** ± **0.08	73.4** ± **7.9	0.72** ± **0.1	0.29** ± **0.08
**GPT-4o Mini**	0.75** ±** 0.05	0.75** ± **0.08	74.5 **± **7.6	0.73 **± **0.1	0.32** ± **0.13
**GPT-4o**	**0.77 ± 0.04**	**0.76 ± 0.07**	**75.9 ± 6.8**	**0.74 ± 0.1**	0.25** ± **0.13
**GPT-4 Turbo**	0.75 **± **0.06	0.76** ± **0.08	75.5 **± **7.5	0.71 **± **0.1	0.30** ± **0.15

MCC: Matthews Correlation Coefficient.

### Performance across items

GPT models, particularly GPT-4o and GPT-4o Mini, reported higher accuracy than other LLMs across four items: feeling depressed (item #2, accuracy = 49.0% and 55.0%), trouble falling or staying asleep, or sleeping too much (item #3, accuracy = 49.0% and 43.0%), tiredness (item #4, accuracy = 36.0% and 40.0%), and psychomotor movements (item #8, accuracy = 72.0% and 68.0%). GPT models were adept at capturing 63.0% of depression symptoms captured by the PHQ-8, particularly those related to emotional states and cognitive functions. The Llama 3 model, however, showed superior performance on questionnaire item #1 (anhedonia) related to having no interest (accuracy = 52.0%), highlighting its effectiveness in detecting anhedonia-related symptoms. Alternatively, the Cohere and Gemini models shared the best performance on the questionnaire item related to appetite (item #5, accuracy = 40.0%), and the Gemini and GPT-4o models performed equally well on the item specifying feeling like a failure (item #6, accuracy = 46.0%).

When binary scores were considered, GPT models continued to have a superior prediction of items related to sleep, tiredness, appetite, and concentration. Llama 3 continued to outperform others on the anhedonia item and showed higher and equal accuracy values (relative to other LLMs) for the items capturing feelings of depression and feeling like a failure (accuracy = 77.0%). The Cohere model was better at predicting the questionnaire item related to psychomotor movement (accuracy = 94.0%). A visual representation of the Likert scale and binary score accuracy results for all LLM models can be found in S1 and S2 Figs respectively.

**Fig 1:**
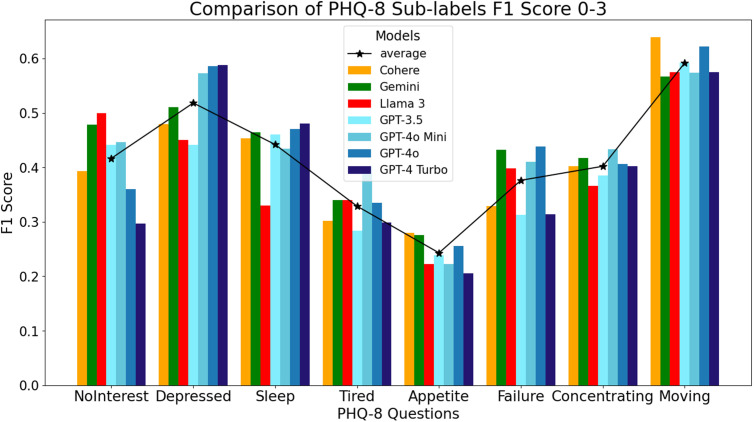
F1 Scores for GPT, Cohere, Llama, and Gemini LLMs for Likert rating scores on individual items of the PHQ-8.

The F1 score analysis for the Likert rating scales (Fig 1) further demonstrates that the GPT models are better for predicting cognitive-affective items of the PHQ-8, particularly feeling depressed, sleep problems, tiredness, feeling like a failure, and concentrating. Within these 5 items, GPT-4 model variants reported higher F1 scores than the GPT-3.5 model. The Llama 3 model maintained its superior performance with respect to anhedonia (F1 = 0.50) and the Cohere model excelled on the items related to appetite (F1 = 0.28) and psychomotor movement (F1 = 0.64). The Gemini model did not excel in any item compared to the other models, although it had comparable F1 scores to the GPT models in predicting sleep, appetite, failure, and concentration.

**Fig 2:**
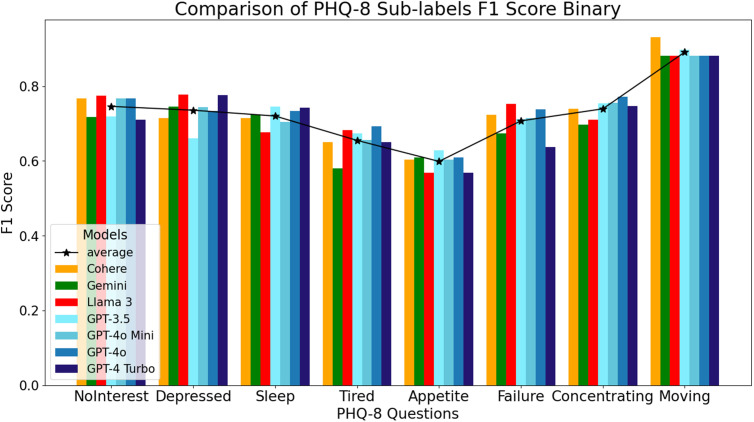
F1 Scores for the GPT, Cohere, Llama, and Gemini LLMs on individual binarized PHQ-8 items.

When looking at binary responses, the GPT models excelled in items related to sleep, appetite, and concentrating, with the Llama 3 model showing higher F1 scores for items reflecting feeling depressed and feeling like a failure (Fig 2). Once again, the Cohere model yielded the highest F1 accuracy score of 0.93 for the psychomotor movement item, even when binary scoring was applied. The Cohere model’s consistent performance on the item related to moving demonstrates a unique advantage in detecting psychomotor symptoms, even in a binarized format.

**Fig 3:**
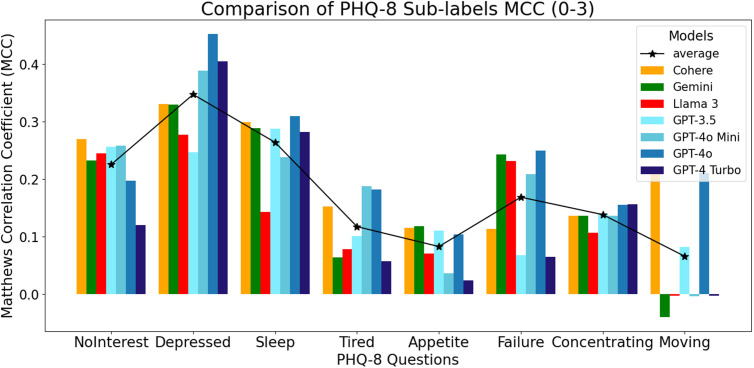
MCC values for individual items on the PHQ-8 scored using a 0-3 Likert rating system.

The MCC results based on the Likert ratings scale scores largely mirrored the accuracy and F1 scores while handling the variability and imbalance in the dataset. As presented in Fig 3, the GPT models continued to perform well on questionnaire items related to feeling depressed, sleep, feeling tired, feeling like a failure, and concentrating when the Likert rating scores were considered. The Cohere model was consistently higher on items related to having no interest and psychomotor movement, and the Gemini model outperformed others on the appetite item. The Llama model did not excel at predicting any PHQ-8 items based on the MCC results.

**Fig 4:**
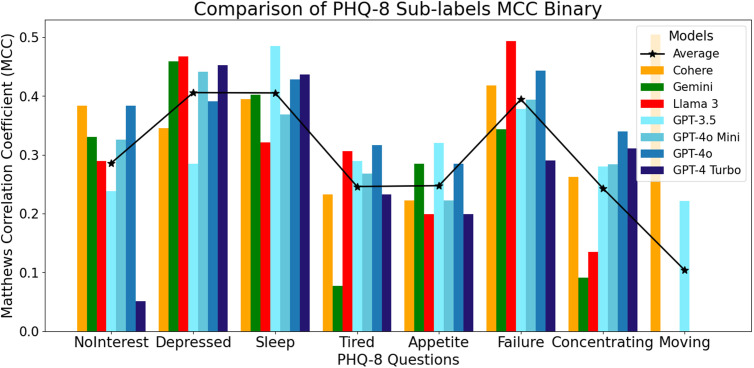
MCC values for individual items on the PHQ-8 scored using a binary system.

When binary scoring was considered, the GPT models excelled in predicting questionnaire items indexing sleep, feelings of tiredness, appetite, and concentrating (Fig 4). The Llama 3 model was more effective on the items of depressed feelings (MCC = 0.47) and feeling like a failure (MCC = 0.49), while the Cohere model remained the best for predicting the psychomotor movement item (MCC = 0.50). Only the GPT-3.5 and Cohere models reported a greater than 0.0 value for the psychomotor movement item.

### Model temperature variation for Llama3

We also explored the impact of different temperature settings on the performance of the Llama3_8B model (Table 2). The default temperature value for the previous experiments was 0.8. There was no clear association between higher accuracy results and increased temperature values. However, temperature settings between 0.6 and 1.4 yielded optimal performance across most items. This range provided a good balance between deterministic and diverse outputs, allowing the Llama 3 model to explore a wider range of possible predictions while maintaining coherence and relevance. A visual representation of these results can be found in the S3 and S4 Figs.

**Table 2:** Average F1 and MMC scores for each PHQ8 item and the respective temperature variations.

F1 Score for Each PHQ8 Item								
Temp. Settings	Non-Interest	Depressed	Sleep	Tired	Appetite	Failure	Concentrating	Moving
**0.0**	0.444	0.462	0.714	0.667	0.250	0.500	0.333	0.0
**0.2**	0.286	0.333	0.750	0.615	0.250	0.400	**0.400**	0.0
**0.4**	0.500	0.429	0.667	0.667	0.250	0.500	0.286	0.0
**0.6**	0.250	0.462	0.667	**0.770**	0.0	0.545	**0.400**	0.0
**0.8**	**0.667**	**0.500**	0.714	0.462	0.0	0.462	0.333	0.0
**1.0**	0.286	**0.500**	0.667	0.667	**0.444**	0.500	0.333	0.0
**1.2**	0.500	0.333	0.714	0.615	0.250	**0.571**	0.333	0.0
**1.4**	0.286	0.462	**0.770**	**0.770**	0.0	0.500	**0.400**	0.0
**1.6**	0.400	0.426	0.667	0.533	0.0	0.429	0.333	0.0
**1.8**	0.286	0.364	0.714	0.462	0.0	0.333	0.333	**0.667**
**2.0**	0.0	0.250	0.500	0.546	0.0	0.0	**0.400**	0.0
**MCC score for Each PHQ8 Item**								
**Temp.** **Settings**	**Non-Interest**	**Depressed**	**Sleep**	**Tired**	**Appetite**	**Failure**	**Concentrating**	**Moving**
**0.0**	0.289	0.218	0.516	0.493	0.293	0.289	0.218	0.0
**0.2**	0.333	0.0	0.500	0.358	0.293	0.378	**0.447**	0.0
**0.4**	0.488	0.149	0.258	0.378	0.293	0.289	0.092	-0.098
**0.6**	0.098	0.218	0.378	**0.618**	0.0	**0.480**	**0.447**	0.0
**0.8**	**0.620**	**0.289**	0.516	0.098	0.0	0.135	0.218	0.0
**1.0**	0.333	**0.289**	0.378	0.493	**0.429**	0.289	0.218	0.0
**1.2**	0.488	0.0	0.516	0.358	0.293	0.258	0.218	0.0
**1.4**	0.333	0.218	**0.674**	**0.618**	0.0	0.289	**0.447**	0.0
**1.6**	0.149	0.149	0.378	0.126	0.0	0.0	0.218	-0.098
**1.8**	0.333	0.073	0.516	0.098	0.0	0.0	0.218	**0.683**
**2.0**	0.0	0.0	0.289	0.364	0.0	0.0	**0.447**	0.0

### Average performance across models

**Fig 5:**
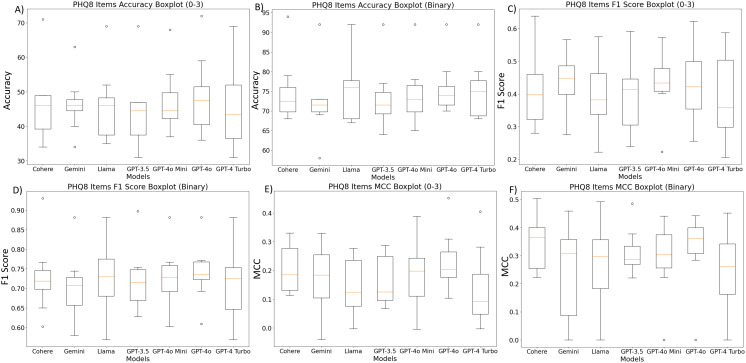
Model comparisons for average values across PHQ-8 items. Panel A and B: Accuracy; Panel C and D: F1 scores; Panel E and F: MCC scores. Likert rating scale scores are depicted on the left of each panel, binary scores are represented on the right.

To facilitate a direct comparison between the models, we calculated the average scores for each performance metric in Fig 5a and 5b for accuracy; (c) and (d) for F1 score; (e) and (f) for MCC across all items. The GPT-4o and GPT-4o mini each present the highest MCC scores across all items and their 4-point Likert scales. However, in the presented analyses, all LLM models performed similarly when considering the average across all items.

## Discussion

This study aimed to evaluate the performance of 7 LLMs from the GPT (GPT-3.5, GPT-4o, GPT-4oTurbo, and GPT-4oMini), Cohere, Llama (v3), and Gemini (v 1.5) families, in predicting mental health (depression) item scores from text transcripts. Both the traditional Likert rating scale system and an adapted binary scoring system were applied. The binarization threshold was selected based on established clinical cut-offs for the PHQ-8, where scores ≥10 are typically indicative of clinically significant depression.8 This threshold aligns with current screening practices and enables evaluation of each LLM’s utility in distinguishing between individuals likely versus unlikely to require clinical follow-up, rather than demarcating symptom severity per se. The primary findings reveal that GPT models (particularly GPT-4o) consistently outperformed others across items conveying emotional states or cognitive functions (i.e., feeling depressed: item 2; sleep: item 3; tiredness: item 4; concentration: item 7). Thus, GPT-4o is a promising candidate for automated mental health screening tools. Alternatively, the strength of the Llama 3 model was in uniquely detecting and predicting anhedonia symptoms (i.e., item 1: having no interest in daily activities), whereas Cohere excelled in identifying and predicting psychomotor activity (item 8). Specifically, the GPT-4o model not only demonstrated better performance in predicting socio-affective PHQ-8 items relative to other out-of-the-box LLMs (Cohere, Llama, and Gemini) but also in relation to other GPT LLMs (-3.5, -Turbo, and -Mini). Although our models demonstrate promising preliminary predictive performance, their interpretability remains a challenge. Clinicians must be able to understand how and why a model arrives at a particular PHQ-8 score to trust and effectively use these tools. Future work should explore the integration of explainability methods, such as attention heatmaps or SHapley Additive explanation (SHAP) values, to provide insight into which linguistic features or response patterns drive model predictions. Enhancing interpretability is critical for clinical adoption and safe integration into practice.

The temperature analysis of Llama 3 models indicated that a temperature range of 0.6 to 1.4 yielded optimal results, suggesting the importance of finding the optimal temperature setting for enhanced model performance. In interpreting the role of temperature in our study, it is crucial to recognize how it enriches our understanding of emotional expression within the interview transcripts. Exploring out-of-the-box decisions, such as integrating temperature as a variable in our model, could enhance its predictive capabilities and lead to more personalized insights. Future refinement studies should consider systematically incorporating temperature in AI-fine tuning alongside other contextual factors, such as situational dynamics and individual differences, to create a more holistic framework for understanding mental health assessments.

The DAIC-WOZ dataset was imbalanced, particularly in the distribution of item scores. Despite using pre-trained LLMs and performing zero-shot classification, the imbalance may shape the reported performance metrics. Accuracy, while a commonly used metric, showed inflated results compared to F1 and MCC metrics (which account for any non-normality or imbalance in the dataset). This was especially true for psychomotor movements (item 8) which yielded a disproportionate number of 0s compared to 1s prior to binarization, leading to a high accuracy score due to the model’s tendency to predict the majority class (scores of 0) more frequently. However, this metric failed to provide a nuanced understanding of the model’s true predictive capabilities, especially in cases where the minority class was of greater clinical relevance. In contrast, the F1 score and MCC offered more reliable insights into model performance. By considering both precision and recall, the F1 score gives a fair demonstration of a model’s performance despite any data imbalance. MCC, with its ability to consider all four confusion matrix categories (hits, misses, false alarms, and correct rejections), provides further insight into the models’ true performances, especially in the presence of imbalanced data. The findings demonstrate that LLMs are capable of screening for depression from textual data in contrast to previous literature that reported inconsistent findings and potential dangers [24].

These findings have important implications for the development of automated screening tools for depression and mental health symptoms more broadly (e.g., stress/distress, anxiety, sleep-related disorders), as well as other medical conditions (e.g., heart disease, cancer). Automated screening tools are effective and useful tools in other areas of healthcare (e.g., congenital heart disease [25]; psychiatric distress [26]). For example, psychiatric distress can be monitored effectively using AI algorithms trained on interview transcripts or social media boards [26]. Although the performance across different LLMs was close, all the GPT models were particularly accurate in the prediction of sleep scores (item 3), which has important implications not only for co-morbid sleep disruptions in depression but also for sleep disorders more broadly. The promising preliminary performance of GPT models across multiple items suggests that these models could be effectively integrated into clinical settings to assist in the rapid and accurate assessment of various mental health symptoms. The variation in model performance across items also highlights the importance of using a combination of models or a model ensemble to capture a holistic evaluation of mental health symptoms. Combining different AI systems to promote more effective outcomes is common practice in other fields of medicine [27]. By leveraging the complementary strengths of various models, an LLM ensemble or “suite” can reduce biases and improve the reliability of predictions across varied symptom presentations. This strategy may enhance clinical decision-making by ensuring a more nuanced and inclusive assessment of mental health conditions, similar to how multimodal diagnostics are utilized in radiology and oncology [28].

When building automated screening models for healthcare, it is important not to *replace* elements of human interaction with AI, but rather to *enhance* the interaction [29]. A collaborative approach with an automated tool augmenting front-line symptom screening and an attending physician confirming mental health diagnoses with standard assessment and diagnostic tools (i.e., Diagnostic and Statistical Manual of Mental Disorders (DSM-5), MINI, MADRS, HAM-D, etc.) can facilitate opportunities for increasing availability of evidence-based insights for health care practitioners to improve patient care and reduce wait-times for screening and referrals [30]. Furthermore, the differences in performance metrics emphasize the need for careful selection of evaluation criteria, especially when dealing with imbalanced and skewed data. While accuracy may provide a quick overview, metrics like F1 score and MCC offer more reliable insights into the true predictive capabilities of the models, particularly in a clinical context where false negatives could have serious consequences.

Finally, the effectiveness of temperature tuning in improving model performance, as demonstrated with Llama 3 models, suggests that further fine-tuning and customization of LLMs could lead to even better outcomes in mental health prediction tasks. This opens avenues for future research focused on optimizing LLMs for specific clinical applications, ultimately leading to more accurate, efficient, and accessible mental health assessments. As well, future research should explore different fine-tuning methods, such as data augmentation and feature engineering, for other mental health symptoms and disorders.

These findings should be interpreted in the context of the inherent subjectivity of both the input data (language) and the labels (survey scores). Rather than seeking to eliminate subjectivity, our approach aims to explore whether LLMs can serve as useful proxies for structured self-report measures in contexts where such instruments may not be administered or where repeated administration may be clinically useful but imposes a burden on patients. The value of LLM-based methods may lie not in exceeding traditional tools in accuracy, but in their scalability, ease of integration into digital platforms, and ability to reduce participant burden. Future work should evaluate the utility of such models in comparison with clinician-rated scales or other behavioural markers of mental health to better contextualize their role in assessment pipelines.

While our findings demonstrate promising LLM performance for PHQ-8 scoring, it is essential to acknowledge the ethical considerations involved in using LLMs for mental health screening. These include potential biases embedded in the training data, which may result in uneven performance across demographic groups, the risk of over-reliance on automated assessments without appropriate clinical oversight, and privacy concerns surrounding sensitive patient data [31]. Future implementations should prioritize transparency, incorporate human-in-the-loop frameworks to ensure clinician oversight, and adhere to rigorous privacy and security standards. Regular model audits and bias mitigation strategies will also be key to the responsible use of these tools.

This study has a few limitations to consider. The accuracy of predictions may be influenced by the quality and diversity of the interview transcripts used. If the dataset lacks representation across different demographics or clinical backgrounds, the model’s performance might not generalize well. We tried to adjust for this using the MCC and F1 scores; however, it is something to consider in future research. As well, the PHQ-8 is intended to assess symptoms over a specific time frame (past two weeks) and is susceptible to change [8]. If transcripts reflect varying timelines or symptoms that fluctuate, this may affect the model’s ability to make accurate predictions. A key limitation of our current approach is its reliance solely on text data, which excludes important non-verbal cues such as tone of voice, speech cadence, and facial expressions. In future work, we intend to explore multimodal models that incorporate audio and/or video inputs to capture these additional layers of information. Such models may improve predictive accuracy and better reflect the complexities of real-world clinical interactions, though they also introduce new technical and ethical considerations. Finally, due to the scarcity of publicly available mental health datasets due to ethical considerations and privacy concerns, we only evaluated the LLM models using one dataset. We intentionally selected the DAIC-WOZ dataset due to several non-negotiable requirements for our modelling approach: the need for (1) publicly accessible data, (2) English-language conversational text, (3) validated clinical labels for depression, and (4) item-level PHQ-8 subscale scores (0–3) for each transcript. To the best of our knowledge, DAIC-WOZ is the only dataset currently available that satisfies all these criteria simultaneously. While this dataset has become a standard benchmark for depression analysis, we acknowledge that using a single source limits the generalizability of our findings. Future work will explore the use of complementary or newly developed datasets that provide comparable granularity in diagnostic annotation to further validate our approach.

In conclusion, this study evaluated the effectiveness of 4 LLMs—GPT (including versions 3.5, 4o, Turbo, and Mini), Llama 3, Cohere, and Gemini—in predicting PHQ-8 items from clinical interview transcripts. GPT models (particularly GPT-4o) had the highest performance metrics across cognitive-affective items (emotional states, cognitive functions, and sleep disturbances), highlighting GPT-4o’s potential as a reliable tool for automated screening of depression symptoms. Alternatively, Llama 3 showed a unique strength in identifying symptoms related to anhedonia and the Cohere model was particularly effective in predicting psychomotor activity. Collectively, these findings highlight the capabilities and potential of LLMs as reliable tools for automated screening of depression symptoms. In conclusion, the robust performance of these LLMs across multiple validated items and metrics, especially GPT-4o, marks a significant step forward in the development of automated depression screening tools. These tools could potentially enhance the efficiency and accessibility of mental health screening, providing valuable support to clinicians and patients in clinical settings. Future research should continue to explore model optimization, including fine-tuning and ensemble approaches, to fully uncover the potential of LLMs in mental health care.

## Materials and methods

### DAIC-WOZ dataset

The DAIC-WOZ dataset is a comprehensive collection of clinical interview recordings and self-report questionnaires designed to assess distress levels related to depression, anxiety, and post-traumatic stress disorder [32–34]. This dataset is available to the research community via open-science portals and includes audio recordings, textual transcripts, and video data from 189 sessions conducted with a human interviewer via video conferencing software.

**Fig 6:**
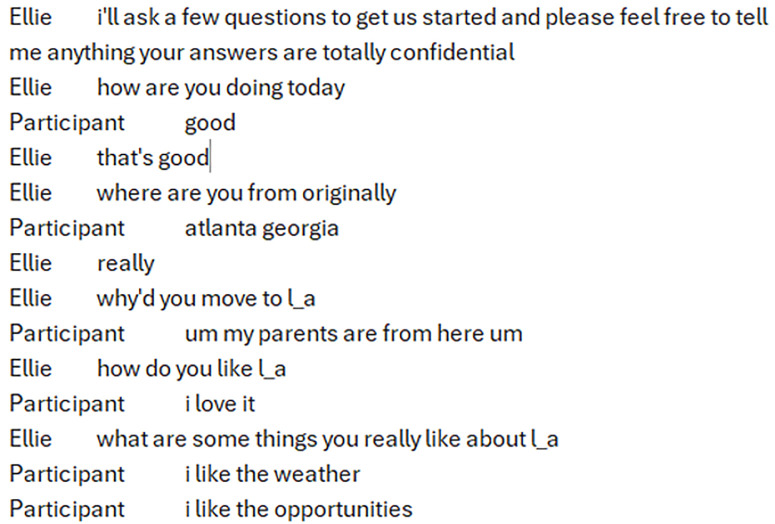
A sample of a transcript from one of the sessions included in the DAIC-WOZ dataset.

The DAIC-WOZ dataset includes semi-structured interviews conducted by a virtual interviewer named Ellie, operated by a human researcher in a Wizard-of-Oz setup. These interviews were designed to collect data related to depression and related conditions. For our study, we randomly selected 100 participants from the DAIC-WOZ set. All personal information in the dataset, such as names, ages, and professions, is either removed or anonymized in the released version, eliminating any risk of personal information exposure. Our analysis was based solely on the de-identified transcripts and associated PHQ-8 scores provided in the dataset documentation. Fig 6 highlights how the textual data of the sessions was presented.

Each record from the DAIC-WOZ dataset contains responses of patients to eight questions (PHQ-8), which are rated on a 4-point Likert scale. For the purposes of this study, only textual interview transcripts and PHQ-8 responses were considered. A definition of the PHQ-8 [8] Likert scale and the eight questions can be found in Table 3.

**Table 3:** The definition of the PHQ-8 prompt, questions, and Likert scale anchors.

**The Eight-Item Patient Health Questionnaire for Depression (PHQ-8)** [8]	
**Main Prompt: Over the last two weeks, how often have you been bothered by the following problems?**	
1. No Interest/Anhedonia	Little interest or pleasure in doing things
2. Depressed	Feeling down, depressed, or hopeless
3. Sleep	Trouble falling or staying asleep, or sleeping too much
4. Tired	Feeling tired or having little energy
5. Appetite	Poor appetite or overeating
6. Failure	Feeling bad about yourself – or that you are a failure or have let yourself or your family down
7. Concentrating	Trouble concentrating on things, such as reading the newspaper or watching television
8. Moving	Moving or speaking so slowly that other people could not have noticed. Or the opposite – being fidgety or restless that you have been moving around a lot more than usual
**Likert Scale Definitions**	
1	“Not at all”
2	“Several days”
3	“More than half the days”
4	“Nearly every day”

#### Textual data preprocessing and cleaning.

The textual data preprocessing and cleaning phase involved a series of steps designed to transform the raw transcript data into a clean and standardized format suitable for input into LLMs. Transcript data was downloaded for each participant (*N* = 189). All text data for each participant’s transcript was combined, creating a cohesive and uninterrupted narrative flow of interviewer questions and patient responses. All transcripts were then combined into a single document to facilitate efficient processing and analysis. Timestamps were manually removed from the transcripts to eliminate unnecessary noise and irrelevant information to the data. Validated mental health outcomes/labels (i.e., PHQ-8 total and item scores) were then integrated with the textual data.

#### Ethics statement.

This study did not require approval from a Research Ethics Board (REB) because it exclusively used publicly available, anonymized data. The DAIC-WOZ database is made available for non-commercial research purposes. No live participants were involved in the current study, and no identifiable information was accessed or utilized. The research complies with all applicable ethical standards for the use of publicly available data.

### Large language models evaluated

A suite of advanced LLMs, well known for their capabilities in natural language processing task models, were selected for this study, including multiple versions of OpenAI GPT, Cohere AI, Llama, and Gemini were selected for this study.

OpenAI GPT is a powerful language model family with the capacity to generate human-like text. Version 3.5 offers a model trained with content up to September 2021. The latest iteration (GPT-4) includes several modules, including GPT4-o, GPT4_Turbo, and GPT4o Mini, each that were trained on over a trillion parameters (https://chat.openai.com/chat). Cohere AI is an advanced LLM trained on a diverse corpus of text adept at understanding and generating human-like responses (https://cohere.com/). Gemini, formerly Bard, is a generative AI chatbot developed by Google (https://gemini.google.com/). The developers of the Gemini 1.5 Flash version state it is optimized for high-speed and cost-effective performance in high-volume tasks. Finally, we experimented with the Llama 3_8B model which offers different parameter sizes and capabilities from the other LLMs (https://ai.meta.com/blog/meta-llama-3/).

### Prompt engineering

#### RISEN framework.

To effectively guide the LLMs in predicting mental health disorder item labels, we employed the RISEN (Role, Instruction, Steps, End goal, and Narrowing) structured prompt engineering framework for each LLM family (GPT, Cohere, Llama, and Gemini). This framework helped us design prompts that provided clear context and instructions to the models without the need to use a more sophisticated architecture such as Retrieval-augmented generation (RAG).

The *role* of the LLM was defined as a mental health screening assistant, tasked with analyzing textual data and providing insights into mental health symptoms and item scores on the PHQ-8 scale. The *instructions* for the LLM were to predict PHQ-8 item scores (Likert or Binary) from interview transcript data. The prediction task was broken down into logical *steps*, encouraging the models to follow a structured thought process. This included highlighting relevant aspects of the textual data (e.g., specific symptoms or sentiments) that aligned with the individual questions of the PHQ-8. Accurate prediction of the individual PHQ-8 items was explicitly stated as the *end goal*. Additional context or constraints were provided, *narrowing* the scope of the prediction task to focus the LLM on relevant responses.

We experimented with different variations of prompts to optimize the performance of the LLMs. This included adjusting the level of detail in the instructions, providing additional examples or context, and varying the prompt length to find the most effective approach for each LLM. The prompt engineering framework is listed in their entirety in Table 4.

**Table 4:** RISEN Framework and Prompt Engineering Technique applied to each LLM model.

RISEN Framework and Prompt Engineering Technique	
**Role**	*Act like you are a psychologist, a mental health disorder professional.*
**Instruction**	*The following is a transcript of a conversation between a psychologist and a patient, which is presented inside the triple backticks ```[transcript]```. Determine if, in the past two weeks, the patient has experienced any of the following symptoms from the Patient Health Questionnaire 8-item scale (PHQ-8), which is a measure of depression severity:* *1. Little interest or pleasure in doing things* *2. Feeling down, depressed, irritable, or hopeless* *3. Trouble falling or staying asleep, or sleeping too much* *4. Feeling tired or having little energy* *5. Poor appetite or overeating* *6. Feeling bad about themselves, feeling like a failure, or feeling that they have let themselves or their family down* *7. Trouble concentrating on things, such as schoolwork, reading, or watching television* *8. Moving or speaking so slowly that other people could have noticed, or the opposite: being so fidgety or restless that they have been moving around a lot more than usual*
**Steps**	*For each of the items in the PHQ-8 questionnaire, determine how often the patient has felt that symptom where the four choices are: 0: Not at All, 1: Several Days, 2: More than Half the Days, 3: Nearly Every Day. Start by explaining where in the transcript the patient indicated that particular symptom from the PHQ-8 questions. Then give a score from the four choices based on the explanation.*
**End Goal**	*In the end, I want you to give me a score between 0 and 3 for each of the PHQ-8 questions.*
**Narrowing**	*After your explanation, give me the response in a table format where the first column will include all the questions of the PHQ-8, and the second column will have a numerical value in the range of 0–3.*

### Model temperatures and performance

#### Model temperatures.

LLMs typically offer the ability to adjust the temperature parameter, which controls the randomness and diversity of the generated responses. We explored different temperature settings of the Llama model, ranging from 0.0 to 2.0, to find the optimal balance between coherent and diverse predictions. Lower temperatures, closer to 0.0, tended to produce more deterministic responses, whereas higher temperatures, closer to the 2.0 maximum evaluated, encouraged exploration and variability.

#### Out-of-the-box performance.

While fine-tuning LLMs is a common practice to adapt them to specific tasks, due to the high resource requirements of this study, we opted to evaluate the out-of-the-box performance of existing models. Our decision was guided by considerations regarding generalizability, accessibility, and ethical considerations [35]. We focused exclusively on zero-shot learning with LLMs to balance performance and practicality, as this methodology significantly reduces training time while optimizing inference time, making it more appropriate for real-world clinical applications where quick diagnoses are critical.

We sought to assess the inherent capabilities of LLMs without task-specific fine-tuning to understand their adaptability to a diverse range of mental health screening interview scenarios. Fine-tuning LLMs often requires substantial computational resources and access to large-scale training data, which may not be readily available to all researchers. Supervised fine-tuning also requires a large amount of data to avoid overfitting which justifies the evaluation of zero-shot learning on out-of-the-box LLMs. Lastly, fine-tuning LLMs on sensitive data, such as mental health transcripts, raises ethical concerns regarding privacy and potential bias amplification.

### Performance metrics and score prediction

#### Performance metrics for imbalanced data.

Recognizing the potential imbalanced nature of the DAIC-WOZ dataset and PHQ-8 reported scores, we selected evaluation metrics that are robust and suitable for imbalanced classification tasks: accuracy, precision, recall, F1-score, and MCC. Accuracy calculates the overall correctness of the model’s predictions, relative to actual scores on the PHQ-8, providing a baseline understanding of performance. The F1-score is the harmonic mean of precision and recall, offering a balanced measure that considers both false positives and false negatives. It is particularly useful when dealing with imbalanced datasets. MCC takes into account true and false positives and negatives, providing a single metric that is suitable even for highly imbalanced datasets. MCC values range from -1 to +1, where +1 represents perfect prediction, 0 indicates random prediction, and -1 represents total disagreement between prediction and observation.

#### Data transformation approaches.

We employed two methods for score prediction to address the imbalanced nature of the item scores: data prediction and binarization. In the data prediction approach, the models were trained to predict the original Likert rating scale scores (0: not at all; 1: several times; 2: most of the time; 3: all of the time) for each item of the PHQ-8 questionnaire. This method provides a fine-grained assessment of depression severity and most closely parallels what is done in clinical practice and research. Alternatively, to simplify the prediction task and focus on identifying the presence/absence of symptoms, item scores were binarized into two categories: 0 (symptom absent: representing Likert scores of 0 and 1) and 1 (symptom present: representing Likert scores of 2 and 3). The binarization threshold was selected based on established clinical cut-offs for the PHQ-8, where scores ≥10 are typically indicative of clinically significant depression [8]. This threshold aligns with current screening practices and enables evaluation of each LLM’s utility in distinguishing between individuals likely versus unlikely to require clinical follow-up.

### Implementation details

#### Experimental setup.

The experiments were conducted using Python as the programming language. We leveraged widely used libraries for model evaluation, and performance metric calculations. The textual data was encoded using standard text preprocessing techniques, and input formatting was adapted to the requirements of each LLM.

#### Computational resources.

A variety of computational resources were utilized for running the experiments. Experiments were conducted using Google Colab Pro, taking advantage of its cloud-based T4 GPU resources and sharing capabilities. Kaggle’s cloud-based environment was also utilized for additional computational support (GPT T4 x 2), particularly for larger models. For accessing OpenAI models (GPT-3.5, GPT-4, etc.), Cohere AI models (c4ai-command-r-plus), Meta Llama models (Meta-Llama-3-8B-Instruct), and Google Gemini models (Gemini-1.5-flash), we utilized their provided application programming interfaces (APIs), leveraging their powerful infrastructure.

## Supporting information

S1 Fig Accuracy Scores for GPT, Cohere, Llama, and Gemini LLMs for Likert rating scores on individual items of the PHQ-8. (TIFF)

S2 Fig Accuracy Scores for the GPT, Cohere, Llama, and Gemini LLMs on individual binarized PHQ-8 items. (TIFF)

S3 Fig Temperature scales for F1 Scores of PHQ-8 binary responses in the Llama 3 model. (TIFF)

S4 Fig Temperature scales for MCC values of PHQ-8 binary responses in the Llama 3 model. (TIFF)
