# Supplementary figures and images for: Leveraging large language models for automated depression screening
Source: PLOS Digit Health. 2025 Jul 28;4(7):e0000943. doi: 10.1371/journal.pdig.0000943 (PMC12303271; doi:10.1371/journal.pdig.0000943)

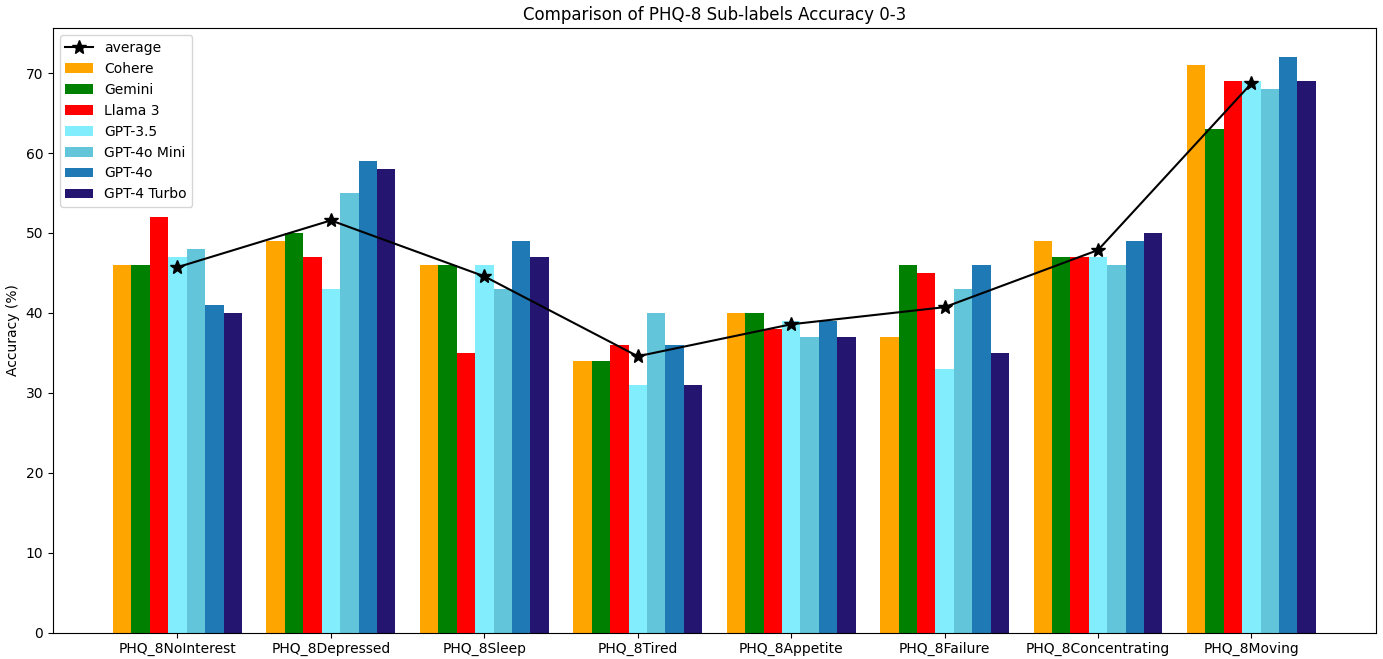

Supplement: S1 Fig — (TIFF) [file pdig.0000943.s001.tiff]

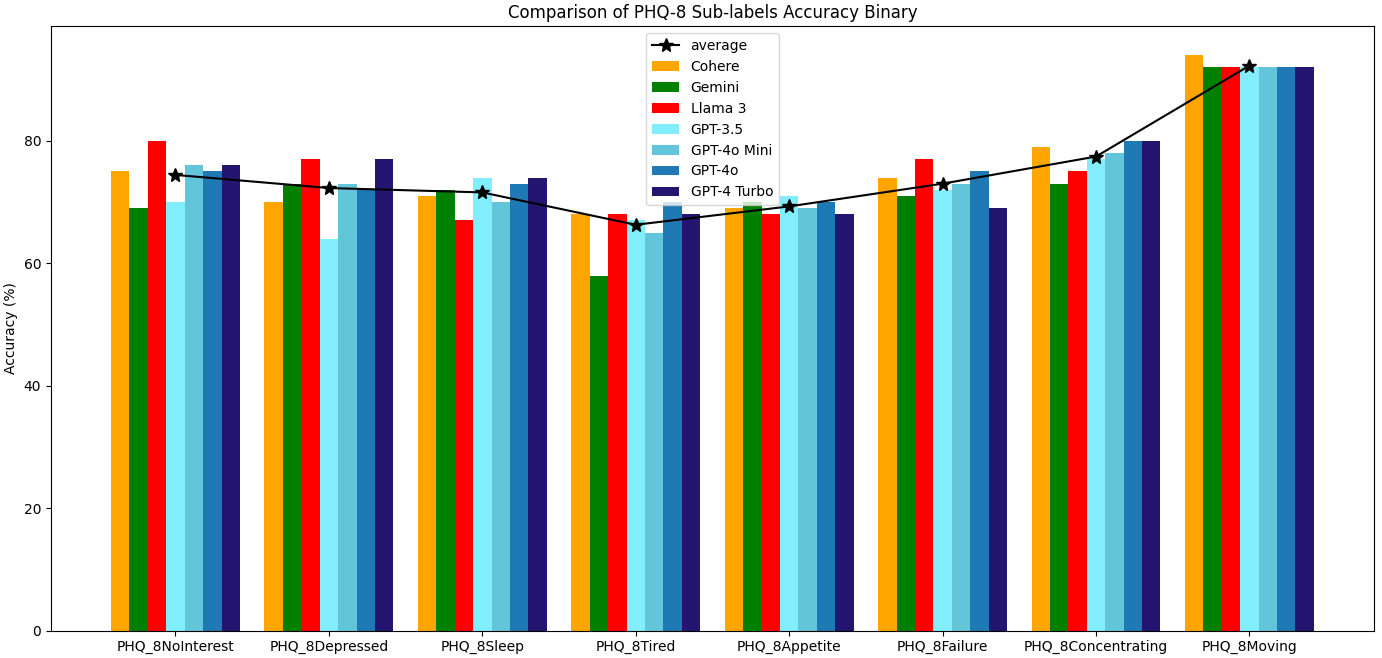

Supplement: S2 Fig — (TIFF) [file pdig.0000943.s002.tiff]

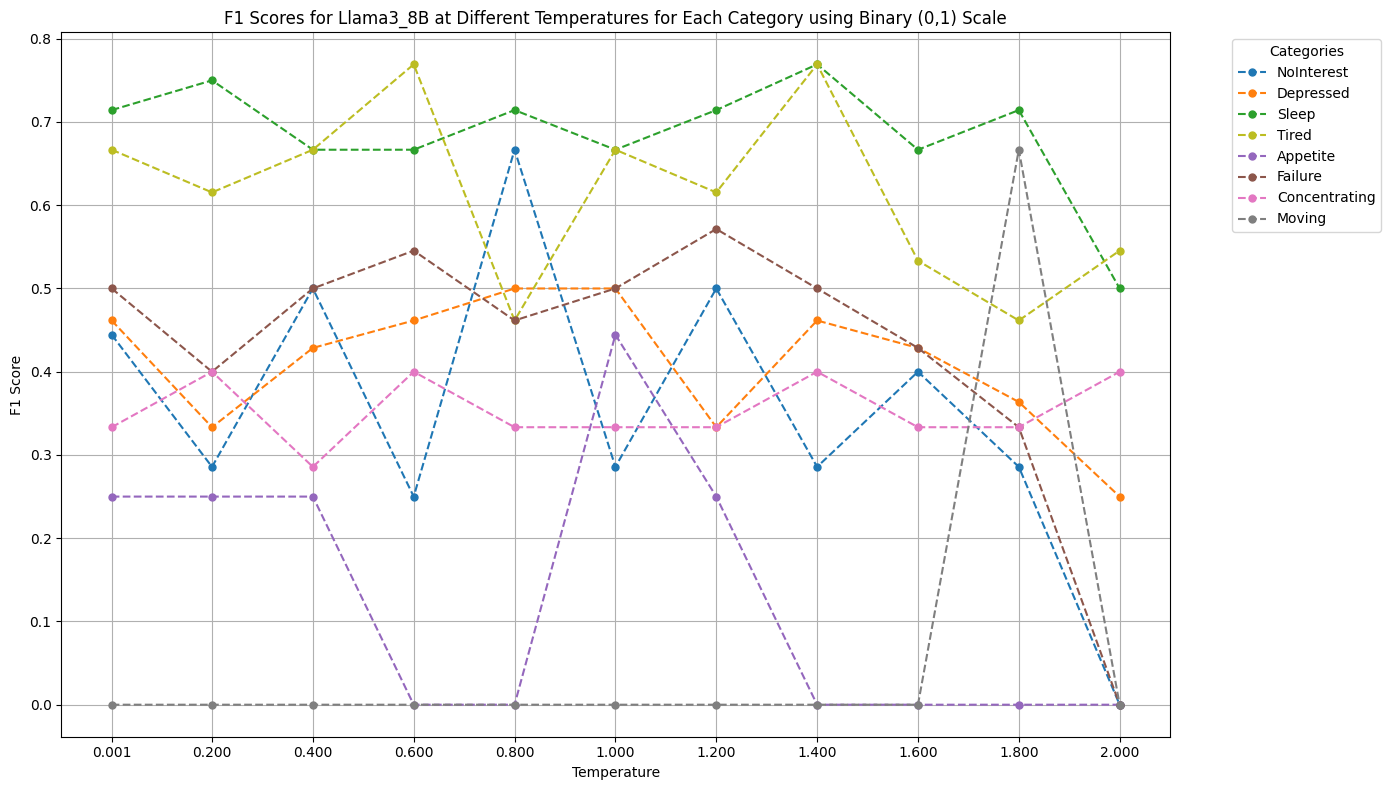

Supplement: S3 Fig — (TIFF) [file pdig.0000943.s003.tiff]

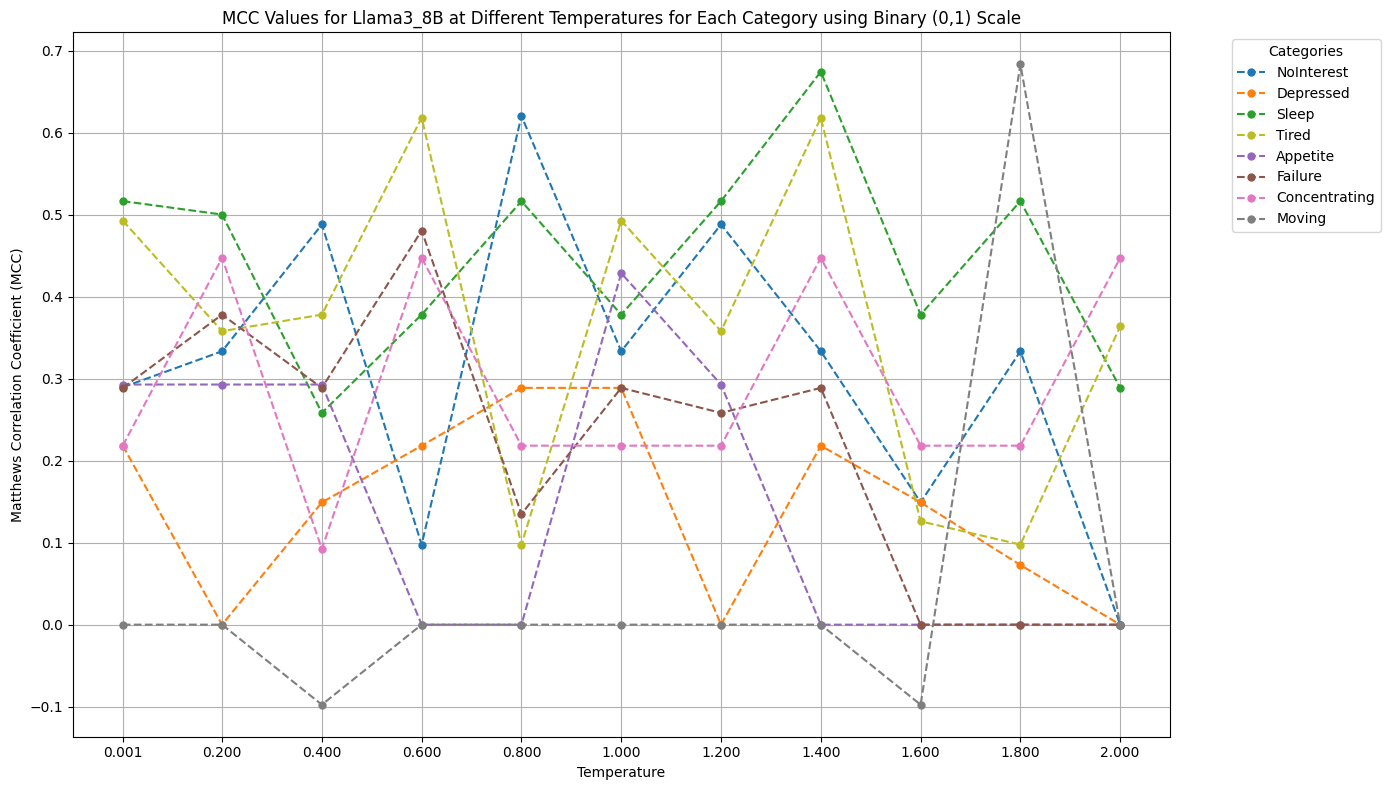

Supplement: S4 Fig — (TIFF) [file pdig.0000943.s004.tiff]
